# A Throughput Request Satisfaction Method for Concurrently Communicating Multiple Hosts in Wireless Local Area Network

**DOI:** 10.3390/s22228823

**Published:** 2022-11-15

**Authors:** Md. Mahbubur Rahman, Nobuo Funabiki, Kwenga Ismael Munene, Sujan Chandra Roy, Minoru Kuribayashi, Melki Mario Gulo, Wen-Chung Kao

**Affiliations:** 1 Graduate School of Natural Science and Technology, Okayama University, Okayama 700-8530, Japan; 2 Department of Informatics and Computer Engineering, Politeknik Elektronika Negeri Surabaya, Surabaya 60111, Indonesia; 3 Department of Electrical Engineering, National Taiwan Normal University, Taipei 106, Taiwan

**Keywords:** Raspberry Pi, WLAN, traffic shaping, access point, fairness, throughput request

## Abstract

Nowadays, the *IEEE 802.11 wireless local area network (WLAN)* has been widely used for Internet access services around the world. Then, the *unfairness* or *insufficiency* in meeting the *throughput request* can appear among concurrently communicating hosts with the same *access point (AP)*, which should be solved by sacrificing advantageous hosts. Previously, we studied the *fairness control method* by adopting *packet transmission delay* at the AP. However, it suffers from slow convergence and may not satisfy different throughput requests among hosts. In this paper, we propose a *throughput request satisfaction method* for providing fair or different throughput requests when multiple hosts are concurrently communicating with a single AP. To meet the throughput request, the method (1) measures the *single* and *concurrent throughput* for each host, (2) calculates the *channel occupying time* from them, (3) derives the *target throughput* to achieve the given *throughput request*, and (4) controls the traffic by applying *traffic shaping* at the AP. For evaluations, we implemented the proposal in the WLAN testbed system with one *Raspberry Pi* AP and up to five hosts, and conducted extensive experiments in five scenarios with different throughput requests. The results confirmed the effectiveness of our proposal.

## 1. Introduction

Currently, the *IEEE 802.11 wireless local area network (WLAN)* has been broadly used around the world for Internet access services [1,2]. The advancements in wireless communication technologies have drastically increased data transmission speeds in WLAN. A WLAN user can access the Internet by connecting with a nearby *access point (AP)* through wireless signals. Then, WLANs have been deployed in governments, companies, schools, and public spaces, with advantages of low-cost installations and flexible area coverages [3,4]. WLAN has become the default media for the Internet access.

However, WLAN cannot guarantee the *fair or request throughput* to every host in the network field. The provided throughput strongly depends on the distance of the host from the AP in the network field. Hence, the fairness issue in WLAN has been widely studied for the *transmission control protocol (TCP)*, since TCP has been adopted in major Internet services such as emails, worldwide webs, and video meetings [5,6].

In fact, our preliminary measurements have revealed that when multiple hosts are concurrently communicating with an AP in WLAN, the *unfairness* or *insufficiency* in meeting the *throughput request* appears among them. In WLAN, a host near the AP receives a higher *received signal strength (RSS)* than a host away from it. Then, the RSS difference will cause the differences in the *TCP congestion window size* and the *modulation and coding scheme (MCS)* at transmitting packets, which will result in the *throughput unfairness* among the hosts.

When a host is located far from the AP, it can suffer from the *insufficient throughput*, although it may need the high throughput to download large files, for example. In this case, the necessary throughput should be allocated to the host by sacrificing the other hosts.

Previously, we studied the *fairness control method* in WLAN. It can achieve fair throughputs among concurrently communicating hosts by controlling *packet transmission delays* of them at the AP using the *PI control* [7]. However, this method suffers from the slow convergence to achieve the fair throughput, since the *delay* is gradually changed by the feedback control of the measured throughput. Besides, it is difficult to satisfy different throughput requests to the hosts, even if necessary.

In this paper, we propose a *throughput request satisfaction method* to solve the drawbacks. This method consists of four steps: (1) it measures the *single throughput* and the *concurrent throughput* for each host, (2) it calculates the *channel occupying time* from the measurement results, (3) it derives the *target throughput* to achieve the request, and (4) it controls the traffic to satisfy the *target throughput* of every host by applying the *traffic shaping* technique at the AP using the *Linux* command *tc*. This technique employs the *Hierarchical Token Bucket (HTB) queuing discipline* [8,9].

The *target throughput* for each host is obtained from the measured *single* and *concurrent throughput* for every host. The *single throughput* gives the average bit rate of the wireless link between this host and the AP. The *concurrent throughput* gives the *channel occupying time* by this link, one per second, when it divides the *single throughput*. The remaining time is occupied by the other links. Then, even if the *concurrent throughput* is replaced by the *target throughput*, this relationship is still true. Based on these observations, the procedure of calculating the *target throughput* for each host is derived.

The goal of the proposed method is to provide the fair or required throughput request among the hosts when they concurrently communicate with a single AP. To achieve the throughput request, the *target throughput* is introduced, which determines how many bits should be transmitted per second by each host. The main contribution of this paper is to present how the *target throughput* is obtained for each host and how it is controlled to achieve the fair or required throughput requests.

- The proper *target throughput* for each host is derived by measuring the single and concurrent throughput and estimating the required *channel occupying time* to satisfy the throughput request.
- Then, the *traffic shaping* is applied using the *Linux tc command* at the *Raspberry Pi AP* to control the traffic of every host to satisfy the *target throughput* without modifying the existing CSMA/CA protocol or the hardware.

For evaluations, we implemented the proposed method in the *WLAN testbed system* using one *Raspberry Pi* AP and up to five hosts. Then, we conducted extensive experiments in five scenarios with different throughput requests. The results confirmed the effectiveness of our proposal.

The rest of this paper is organized as follows: Section 2 discusses the related works. Section 3 reviews our preliminary works. Section 4 presents the throughput request satisfaction method. Section 5 and Section 6 evaluate the proposal through experiments. Section 7 concludes this paper with future works.

## 2. Related Works

In this section, we discuss related works in literature. A number of research works have addressed the TCP unfairness problem in WLAN.

In [10], Kim et al. investigated the asymmetric behavior between the uplink and downlink TCP flows. They designed an adaptive backoff algorithm by estimating the backlog size (number of nodes that have packets) for the uplink/downlink to achieve fairness and optimize the throughput. The ideal uplink and downlink transmission probabilities are derived based on the backlog estimation as a function of the backlog size. The effectiveness is verified through simulations. In contrast, our proposal solves the throughput unfairness problem among the hosts and provides the necessary throughput. The proposal is implemented at the AP by installing conventional Linux commands.

In [11], Priya and Murugan studied the unfairness problem for simultaneous uplink and downlink TCP flows by considering the optimum queue selection. They designed a two-queue approach where the primary queue holds the TCP data packets while the secondary queue holds acknowledgment (ACK) packets. In this method, the optimal queue size is identified by the probability or priority scheduling approach. In the priority scheduling, the ACK packets are given higher priority and are transmitted before the data packets. In the probability scheduling, the AP selects the queue based on the optimal probability *p* to ensure fairness, where *p* is calculated considering the number of the uplink and downlink flows. They implemented it by the modification of the MAC layer protocol and verified it through simulations. In contrast, we study the fair or required throughput among the competing hosts, instead of uplink/downlink fairness, which can be implemented on a real testbed system without modifying the MAC protocol.

In [12], Kim et al. examined the throughput unfairness problem in WLAN that is caused by *unequal frame error rates (FERs)* among hosts and the absence of loss differentiations in the *automatic repeat request (ARQ)* protocol, which can lead to the imbalance of the outage probability and the access probability among hosts. The authors proposed the *enhanced distributed coordination function (DCF)* by adopting the *hybrid automatic repeat request (HARQ) with Chase combining (HARQ-CC)* to solve both imbalance problems. The performance of the method is demonstrated both mathematically and through MATLAB simulations. However, for the practical implementation, the Media Access Control (MAC) layer protocol needs to be modified. On the other hand, our proposal can be implemented by calling the Linux commands from an application program. It does not need modifying the MAC protocol implementation.

In [13], Lei et al. studied the airtime fairness in WLAN. They presented the *improved active queue management (IAQM)* algorithm for solving the unfairness problem of WLAN by setting the different queue lengths based on their data rates so that each host gets the fair channel usage time. In contrast, our proposal achieves throughput fairness using the traffic control command for traffic shaping.

In [14,15], Kongsili et al. and Fang et al. addressed the unfair channel access time in wireless networks when a device communicates at a low data rate. Kongsili et al. proposed an algorithm for overcoming the unfairness problem by integrating the channel access priority control and packet scheduling. The proposal was implemented at the AP and was verified through simulations. Fang et al. introduced a method for the airtime control strategy by using the *Hierarchical Token Bucket (HTB)* bandwidth management and verified through testbed experiments. These approaches enhance the network throughput by ensuring fair airtime to the hosts, but there is still an unfairness when considering the equal throughput performance of the hosts. In contrast, our proposal ensures the throughput fairness or the required throughput among the competing hosts, where the effectiveness is verified through real testbed experiments.

In [16], Mansy et al. introduced a new *quality of experience (QoE)* metric to ensure the network layer fairness for adaptive video streams. A max-min fairness problem is devised based on this metric to enforce bandwidth allocations in the home network, and the traffic shaping is applied to control network traffic. In contrast, our proposal allocates the equal or required throughput by assigning the proper target throughput to the host, where the effectiveness is verified through testbed experiments.

In [17], Hwang et al. studied the unfairness problem in a multi-rate WLAN. They observed that the throughput performance of a network is drastically degraded due to the excessive channel use by low-rate clients. Hence, they proposed a network-wide association scheme with a traffic allocation method that can boost the network throughput while maintaining fairness. Traffic is controlled by traffic shaping. The proposal was verified by simulations. In contrast, our proposal is implemented using the Linux command at the AP and is verified through testbed experiments.

In [18], Høiland-Jørgensen et al. introduced a network layer queue management scheme to ensure fairness among the competing hosts in WLAN. The proposal eliminates the performance anomaly of the wireless network and improves the overall throughput. It was implemented at the AP with no modification at the MAC layer protocol.

In [19], Le et al. proposed a method to solve the unfairness problem by allowing each station to choose an appropriate contention window size based on the cost function. They implemented it in the MAC layer and verified it through simulations. In contrast, our proposal is implemented in a real testbed system adopting the traffic shaping at the AP to achieve the throughput fairness.

In [20], Garroppo et al. observed that the performance of the 802.11 standard is severely degraded when a single station experiences poor channel condition to the AP. This performance anomaly occurs due to the simple FIFO scheduling manner employed in the AP and the max-min fairness of the CSMA/CA protocol. In order to overcome this problem, they proposed the *Deficit Transmission Time (DTT)* scheduler to ensure fair airtime usage to all the associated stations. The *Wireless Channel Monitor (WChMon)* tool is used to estimate the maximum attainable throughput towards the specific station. However, the major drawback of this tool is the dependence on the specific network card and driver. In contrast, our proposal considers throughput fairness instead of time fairness and is not dependent on the specific network card and driver.

In [21], Blough et al. dealt with proportional fairness to improve the overall network throughput by considering the *signal to noise ratio (SNR)* level at receiving stations. The SNR is used in their approach to determine the appropriate data transmission rate based on the channel condition to ensure fairness among the competing hosts. Hosts with high transmission rates are allowed to transmit more packets compared to hosts with low transmission rates. In contrast, our proposal deals with the per-host throughput fairness and the required throughput among the competing hosts.

In [22], Banchs et al. introduced an algorithm to ensure throughput fairness in virtual WLANs by using the control theory. This proposal adopted the proportional integrator (PI) controller to adjust the contention window of each virtual WLAN to achieve optimal performance. The effectiveness of this method is verified by simulations. However, in reality, to control the contention window is difficult where hardware modification is required. Besides, this method cannot ensure equal or required throughput among the concurrently communicating hosts. In contrast, our proposal uses traffic shaping at the AP to allocate the equal or required throughput to the hosts without modifying the hardware.

In [23], Akimoto et al. observed that the locations of *mobile terminals (MTs)* in the network result in different coverages, where some terminals may cause the hidden terminal problem. This problem degrades the throughputs of the affected terminals while others have high throughputs. To address this issue, the authors proposed the mobile terminal allocation scheme using the virtual sector (VS) where terminals are classified into groups by their coverages. Terminals in one group can sense each other during data transmissions to avoid the hidden terminal problem and solve the throughput unfairness. However, our experiment results show that the throughput unfairness is observed even if stations do not suffer from the hidden terminal problem, when they communicate from different relative distances from the AP. In addition, their approach is evaluated throughput simulation and required to modify the MAC layer protocol.

In [24], Abuteir et al. presented a *software-defined networking (SDN)* based *wireless network assisted video streaming (WNAVS)* framework to ensure the proportional fairness among the users. The proposal applies the traffic shaping to control the data packets based on the throughput allocations to the users. Their method is limited to a specific application such as video and cannot satisfy the equal or necessary throughput request among the hosts.

Table 1 shows the comparisons between our proposal and 15 approaches in the literature. Most of the existing approaches in our literature review focus on airtime fairness or fairness between the uplink/downlink flows. In airtime fairness, the proper airtime is assigned to the hosts based on the data rates in WLAN. On the other hand, the different queuing techniques are used to ensure fairness between uplink/downlink flows. However, While this strategy can improve the overall network throughput, the unfairness issue remains among the hosts, when considering equal throughput performances. Besides, these approaches cannot meet the required throughput requests of the host when they are concurrently communicating with the AP from different relative distances. To address the issues, we propose a *throughput request satisfaction method* to ensure the throughput fairness or the throughput request. We adopt the network-level queuing approach that allows the AP to control the packets according to the target throughputs of the hosts. In terms of target throughput, it refers to how many packets per second a host is supposed to transmit, which is derived from the measured single and concurrent throughput for every host.

The IEEE 802.11e is designed to enhance the 802.11 MAC to guarantee the Quality of Service (QoS) in WLANs [25]. It introduces *Enhanced Distributed Channel Access (EDCA)* and *Hybrid Coordination Function (HCF)*, which provides traffic differentiation and priorities, but the achievable throughput can be extremely low, and the performance obtained is not optimal, since EDCA parameters cannot be properly adjusted according to the network conditions. Our study focuses on the 802.11n, which has the advantage of providing a higher throughput performance than 802.11e [26]. However, 802.11n cannot ensure fair throughput among the competing hosts. Therefore, we propose the throughput request satisfaction method that ensures fairness among the hosts.

Our proposal uses the conventional Linux command *tc* to apply the *traffic shaping* to control the traffic at the AP, which can be easily implemented at the application program.

## 3. Preliminary

In this section, we briefly introduce preliminary studies and technologies to this paper.

### 3.1. Throughput Unfairness Observation

**Figure 1:**
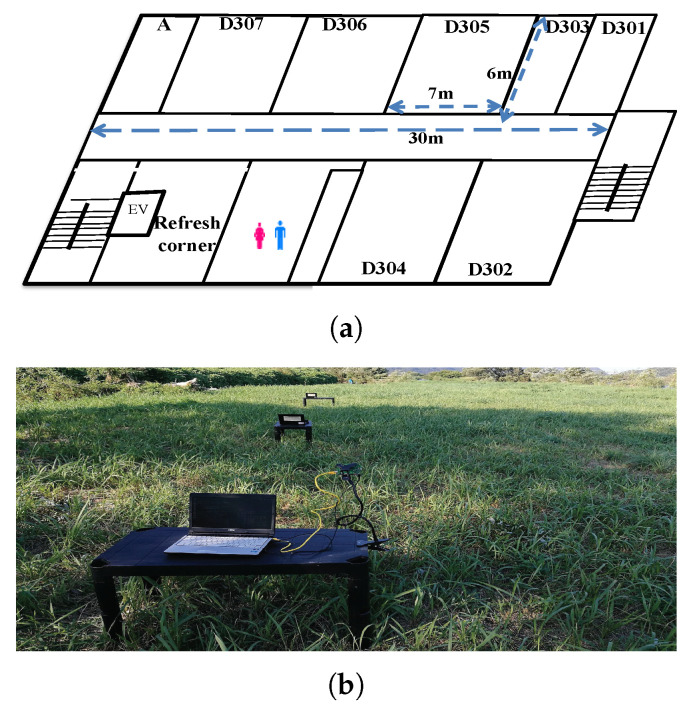
Experiment fields. (**a**) Indoor environment. (**b**) Outdoor environment.

In WLAN, the throughput unfairness may appear among the hosts when they concurrently communicate with the same AP at different relative distances. Previously, we performed throughput measurements of concurrently communicating two hosts with the same AP in the corridor of Engineering Building #2 in Okayama University (indoor environment) and Asahi riverbed (outdoor environment). Figure 1a illustrates the experiment field for the indoor environment where interferences from other WLANs exist, while Figure 1b does the outdoor environment without any interference. In both fields, the hosts communicate with the *Raspberry Pi* AP using the *IEEE802.11n* 20 MHz channel at 2.4 GHz.

**Figure 2:**
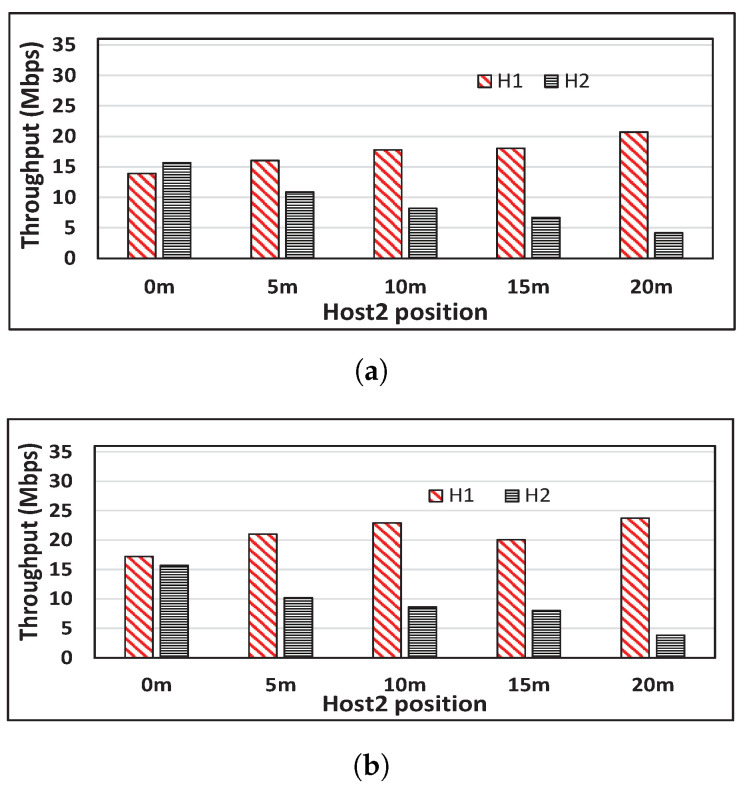
Throughput unfairness observations between two hosts. (**a**) Indoor result. (**b**) Outdoor result.

In our experiments, we used a single spatial stream, which supports the *modulation and coding schemes (MCS)* index values from 0 to 7 [27]. The host $H_{1}$ is fixed at 0 m distance from the AP, and the host $H_{2}$ is moved from 0 m to 20 m with the 5 m interval from the AP. Figure 2 shows that the throughput difference between the two hosts increases as the distance between $H_{2}$ and the AP increases, and that the throughput of $H_{1}$ increases, although the location is fixed in both network fields.

### 3.2. Saturated Host

A host may be connected to a server on the Internet that needs a small throughput for the application, offers a small processing capability, or has a small bandwidth section. Then, the achieved throughput of the host can be saturated and be smaller than the fair throughput in the WLAN. For example, the popular video meeting service *zoom* requires 2 Mbps for the single screen [28], which is much smaller than the available bandwidth of IEEE 802.11n WLAN.

In this paper, this host is called the *saturated host*, and the maximum achieved throughput is the *saturated throughput* for convenience. To avoid wasting the limited bandwidth in WLAN, the saturated throughput should be assigned to the *saturated host*, and the remaining bandwidth be shared among the other hosts.

### 3.3. Traffic Shaping

*Traffic shaping* allows us to control the network bandwidth by scheduling, policing, shaping, and classifying the network traffic, to provide the guaranteed bandwidth service for the specific user. In *Linux*, *traffic control (tc)* command can be used for *traffic shaping*. There are three components for the *tc* command, namely, *queueing discipline (qdisc)*, *classes*, and *filters*. The *qdisc* scheduler is categorized into two groups of *classless qdisc* and *classful qdisc*. The *classful qdisc* permits us to categorize traffic that demonstrates different treatments. In contrast, the *classless qdisc* does not allow us to classify the traffic.

In this paper, we adopt the *classful HTB qdisc* to control the traffic at a specific rate. The *HTB* uses *token buckets* for the link-sharing classes. Each class contains two parameters, *ceil* and *rate*, to specify the amount of traffic allocated to each class. The *rate* refers to the guaranteed bandwidth of the whole class and the *ceil* refers to the maximum bandwidth of each traffic. In this paper, we give the same value to them.

### 3.4. Throughput Measurement Tool

In this paper, *iftop* [29] is installed at the AP as the open-source network traffic monitoring tool to measure the throughput for each host. *iperf* [30] is also used to generate traffic required for the throughput measurement using *iftop*.

## 4. Throughput Request Satisfaction Method

In this section, the *throughput request satisfaction method* is presented for the hosts that are communicating concurrently with a single AP.

### 4.1. Observations of Proposal

The following observations are considered in designing the proposed method.

(1) The *traffic shaping* can control the throughput of each host by applying *tc command* at the AP.
(2) The *single throughput* for each host can be measured using *iftop* when only one host communicates with the AP, which can give the average *maximum number of transmitted bits per second (bps)*.
(3) The *concurrent throughput* for each host can be measured using *iftop* when all hosts communicate concurrently with the AP, which can give the average *actual number of transmitted bits per second (bps)*.
(4) The *target throughput* refers to the number of bits transmitted per second by each host.
(5) The *concurrent throughput* and *single throughput* for each host can be used to estimate the average *channel occupying time* per one second. The *concurrent throughput* is divided by the *single throughput* to obtain the average *channel occupying time*. Then, this relationship is still true if the *concurrent throughput* is substituted by the *target throughput*.
(6) The total of the average *channel occupying time* for all the hosts can be constant (basically, one second).
(7) The *single throughput* of the host is always higher than the *target throughput*.
(8) When the *target throughput* of a host exceeds the concurrent one, the time allocated to this host can be increased by taking the *channel occupying time* of the other hosts. Thus, a proper target throughput for each host can be determined.

### 4.2. Single and Concurrent Throughput Measurement

In the proposed method, first, the *single throughput* and the *concurrent throughput* for every host is measured at the target AP, to calculate the proper target throughput for each host. The *single throughput* is measured for each host by limiting only the host to communicate with the AP. The *concurrent throughput* is measured by activating all the hosts to communicate with the AP.

### 4.3. Channel Occupying Time of Hosts

The notations used in this paper are described in Table 2. When the hosts $H_{1}$, $H_{2}$, ..., $H_{n}$ share the same channel during concurrent communication, they occupy the channel for a certain period of time to transmit the data. Therefore, the average *channel occupying time* per one second for each host can be estimated by $\frac{C_{1}}{S_{1}}$, $\frac{C_{2}}{S_{2}}$, ..., $\frac{C_{n}}{S_{n}}$ and their sum will be constant, as follows:

(1) $$\frac{C_{1}}{S_{1}}+\frac{C_{2}}{S_{2}}+...+\frac{C_{n}}{S_{n}}=Constant$$

The *CSMA/CA protocol* in the WLAN activates the wireless links between the AP and the associated multiple hosts in turns. It basically repeats the *data transmission* of one host through the channel and the *channel idling* for the contention resolution.

During the unit time of one second, the average *data transmission time* of the link with the host $H_{i}$ can be estimated by $\frac{C_{i}}{S_{i}}$, because $C_{i}Mbit$ data is transmitted through the $S_{i}Mbps$ link. The *channel idling time* can be constant when the number of the contending hosts is constant, because each contention resolution time in the CSMA/CA protocol can be constant on average.

To achieve the throughput request, the proposed method does not change the number of contending hosts. It only changes the *data transmission time* of links while keeping their communications. As a result, the *channel idling time* is not changed before and after applying the proposed method. Thus, for simplicity, the *channel idling time* is neglected in this equation.

### 4.4. Equal Target Throughput Request

First, we discuss the calculation of the *target throughput* when all the hosts are assigned the same target throughput: $t_{1}$ = $t_{2}$ = ... = $t_{n}$. Then, to transmit $t_{1}$, $t_{2}$, ..., $t_{n}$Mbit data through $S_{1}$, $S_{2}$, ..., $S_{n}$ link, the *channel occupying time* for the hosts will be $\frac{t_{1}}{S_{1}}$, $\frac{t_{2}}{S_{2}}$, ..., $\frac{t_{n}}{S_{n}}$.

#### 4.4.1. Conventional Host Case

When there is no saturated host in the WLAN, the following result is obtained from Equation (1):

$$\frac{C_{1}}{S_{1}}+\frac{C_{2}}{S_{2}}+...+\frac{C_{n}}{S_{n}}=\frac{t_{1}}{S_{1}}+\frac{t_{2}}{S_{2}}+...+\frac{t_{n}}{S_{n}},$$

(2) $$t_{1}=t_{2}=...=t_{n}=\frac{\sum_{\begin{array}{c} i=1 \end{array}}^{n}\frac{C_{i}}{S_{i}}}{\sum_{\begin{array}{c} i=1 \end{array}}^{n}\frac{1}{S_{i}}}.$$

#### 4.4.2. Saturated Host Case

If the saturated host (let $H_{k}$) exists in the WLAN where the derived target throughput is larger than its *single throughput* $S_{k}$, the target throughput for each host is updated by the following procedure to avoid the bandwidth waste:

$$t_{k}=S_{k},$$

(3) $$t_{1}=t_{2}=...=t_{n}=\frac{1}{\sum_{\begin{array}{c} i=1\\ i\neq k \end{array}}^{n}\frac{1}{S_{i}}}\left(\sum_{\begin{array}{c} i=1 \end{array}}^{n}\frac{C_{i}}{S_{i}}-1\right).$$

### 4.5. Different Target Throughput Request

Next, we discuss the calculation of the target throughput for $H_{2}$, $H_{3}$, ..., $H_{n}$ when the different target throughput $t_{1}$ of $H_{1}$ should be satisfied. Here, $t_{1}$ must not be larger than the *single throughput* $S_{1}$ and must not be smaller than the *minimum target throughput* $t_{min}$. In this paper, the *minimum target throughput* is introduced to guarantee the least throughput for any host, even if some host asks for a very high target throughput. Then, since another equation is necessary to give the unique values of $t_{2}$, $t_{3}$, ..., $t_{n}$ for the given $t_{1}$, the equal target throughput is assumed for their fairness: $t_{2}$ = $t_{3}$ = ... = $t_{n}$.

#### 4.5.1. Conventional Host Case

When there is no saturated host in the WLAN, the following result is obtained from Equation (1):

$$\frac{C_{1}}{S_{1}}+\frac{C_{2}}{S_{2}}+...+\frac{C_{n}}{S_{n}}=\frac{t_{1}}{S_{1}}+\frac{t_{2}}{S_{2}}+...+\frac{t_{n}}{S_{n}},$$

(4) $$t_{2}=t_{3}=...=t_{n}=\frac{1}{\sum_{\begin{array}{c} i=2 \end{array}}^{n}\frac{1}{S_{i}}}\left(\sum_{\begin{array}{c} i=1 \end{array}}^{n}\frac{C_{i}}{S_{i}}-\frac{t_{1}}{S_{1}}\right).$$

#### 4.5.2. Saturated Host Case

If the saturated host (let $H_{k}$) exists in the WLAN where the derived target throughput is larger than its *single throughput* $S_{k}$, the target throughput for each host except $t_{1}$ is updated by the following procedure to avoid the bandwidth waste:

$$t_{k}=S_{k},$$

(5) $$t_{2}=t_{3}=...=t_{n}=\frac{1}{\sum_{\begin{array}{c} i=2\\ i\neq k \end{array}}^{n}\frac{1}{S_{i}}}\left(\sum_{\begin{array}{c} i=1 \end{array}}^{n}\frac{C_{i}}{S_{i}}-1-\frac{t_{1}}{S_{1}}\right).$$

#### 4.5.3. Minimum Target Throughput Case

If the derived target throughput for $H_{2}$, $H_{3}$, ..., $H_{n}$ becomes smaller than the *minimum target throughput* $t_{min}$, the target throughput for every host is updated by the following procedure to ensure it.

If $S_{k}$ < $t_{min}$, $t_{k}$ = $S_{k}$, and use the following equation to updates the target throughput to ensure $t_{2}$ = $t_{3}$ = ... = $t_{n}$ = $t_{min}$.

(6) $$t_{1}=\sum_{\begin{array}{c} i=1 \end{array}}^{n}\frac{S_{1}C_{i}}{S_{i}}-S_{1}-\left(\sum_{\begin{array}{c} i=2\\ i\neq k \end{array}}^{n}\frac{t_{min}S_{1}}{S_{i}}\right).$$

Otherwise, updates the target throughput as follows:

$$t_{2}=t_{3}=...=t_{n}=t_{min},$$

(7) $$t_{1}=\sum_{\begin{array}{c} i=1 \end{array}}^{n}\frac{S_{1}C_{i}}{S_{i}}-\left(\sum_{\begin{array}{c} i=2 \end{array}}^{n}\frac{t_{min}S_{1}}{S_{i}}\right).$$

### 4.6. PI Controller for Rate and Ceil Parameter

In *traffic shapping*, the *rate and ceil* parameter value $d_{i}$ can control the maximum bandwidth of the host at communications. Unfortunately, it does not guarantee the given specific throughput. Thus, the measured throughput will be fluctuating during communications. To overcome this limitation, the *PI feedback control* [31,32] is introduced to make the measured throughput equal to the target one by dynamically updating $d_{i}$. The updated value of $d_{i}$ must be greater than or equal to the $t_{i}$. In the system implementation, the following equation is adopted:

(8) $$\;d_{i}\left(m\right)=d_{i}\left(m-1\right)+K_{P}\times \left(R_{i}\left(m-1\right)-R_{i}\left(m\right)\right)+K_{I}\times \left(t_{i}-R_{i}\left(m\right)\right).$$

Equation (8) is applied when the throughput error $|R_{i}\left(m\right)-t_{i}|$ exceeds a certain threshold $\alpha \times t_{i}$ during three consecutive time steps (one-time step equals to 60 s) to prevent frequent changes of $d_{i}$. Here, $R_{i}\left(m\right)$ is obtained by measuring the throughput at every time step and α=0.2 defines the constant parameter. In this paper, $K_{p}=0.4$ and $K_{I}=0.5$ are used as the PI control parameters.

### 4.7. Application of Traffic Shaping

In our implementation of the AP using *Raspberry Pi*, traffic shaping is applied using *tc* command with the following procedure:

(1) Create the *HTB qdisc*, generate the required number of classes for each host *i*, and assign the *rate* value $d_{i}$ by:
  – $sudo tc qdisc add dev wlan0 root handle 1: htb default.
  – $sudo tc class add dev wlan0 parent 1: classid 1:1 htb rate $\sum_{i=1}^{n-1}d_{i}$
  – $sudo tc class add dev wlan0 parent 1:1 classid 1:*i* htb rate $d_{i}$ ceil $d_{i}$.
(2) Apply the $d_{i}$ to the host $H_{i}$ by specifying the IP address:
  – $sudo tc filter add dev wlan0 protocol ip parent 1:0 prio 1 u32 match ip dst IPof$H_{i}$ flowid 1:*i*.

### 4.8. Procedure of Throughput Request Satisfaction Method

**Figure 3:**
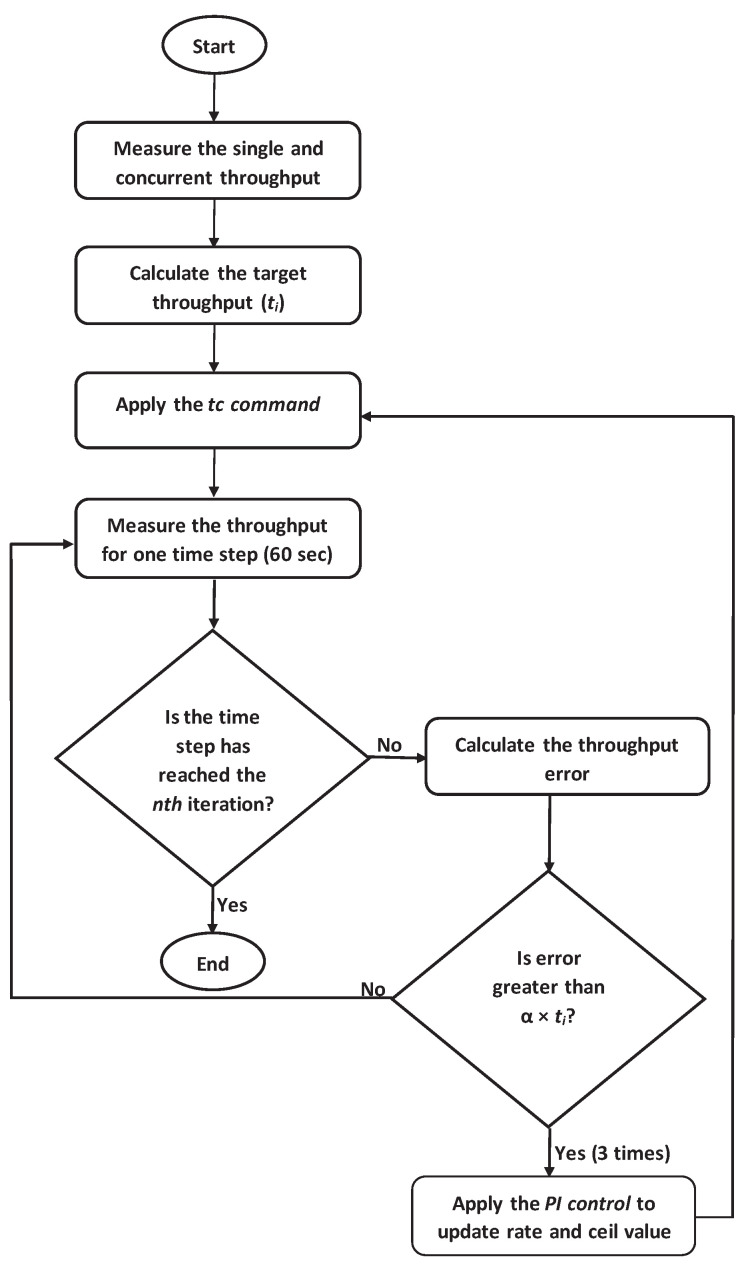
Flow of throughput request satisfaction method.

Figure 3 illustrates the flow of the whole procedure in the proposed method. The following procedure describes the application of the proposed method.

(1) Measure the *single throughput* for each host using *iftop* while only the host is communicating.
(2) Measure the *concurrent throughput* for every host using *iftop* while all the hosts are communicating simultaneously.
(3) Calculate the *target throughput* and assign the initial *rate and ceil* value by $d_{i}$ = $t_{i}$.
(4) Apply the *traffic shaping* using *tc*.
(5) Periodically measure the *concurrent throughput* for every host using *iftop* while all the hosts are communicating.
(6) Apply the *PI control* to update $d_{i}$.

## 5. Evaluations with * iperf* Traffic

In this section, we evaluate the proposal through testbed experiments using *iperf* traffic with up to five hosts.

### 5.1. Experimental Setup

**Figure 4:**
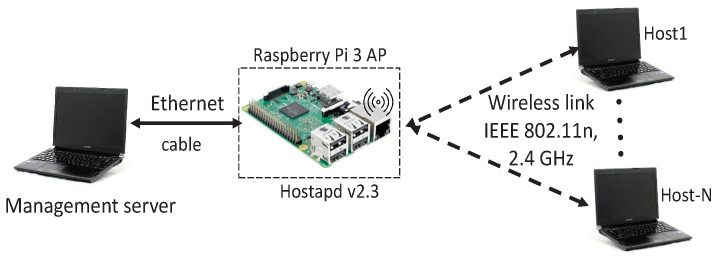
Testbed topology for *iperf* traffic.

Figure 4 and Table 3 show the network topology and the hardware/software specifications of the testbed system respectively. *Raspberry Pi 3* is used as the software AP and the *Linux-based* PCs are for the hosts and the management server. Table 4 shows the locations of the AP and the hosts in the experiments where the indoor field in Figure 1a was used.

The measured throughput often fluctuated. To improve measurement of the accuracy, the throughput measurement for each scenario was repeated 12 times and their average result was used in evaluations. One measurement took one minute. Thus, the total measurement time for each scenario was 12 min.

### 5.2. Experiment Scenarios

In our experiments, the five scenarios on target throughput conditions in Table 5 were considered. In any scenario, the same TCP traffic was generated using *iperf 2.0.5 software* with 477 KB TCP window size and 8 KB buffer size. In this paper, $t_{min}=1.5$ Mbps was used for Table 5.

*(1) Equal Throughput:* All the hosts are assigned the same throughput. This scenario intends to examine the throughput fairness request among the hosts.

*(2) High Priority Host A:* The fastest host $H_{1}$ is considered as the *high priority host* and is assigned a higher target throughput than the other hosts that are assigned the same throughput. This scenario intends to examine the simultaneous requests of the high throughput and the fairness among the hosts.

*(3) High Priority Host B:* The same throughput setup is considered here except for the condition that the original target throughput by the proposal does not meet the minimum target throughput. Thus, *Minimum Target Throughput Case* in Section 4.5.3 is applied here.

*(4) Low Priority Host A:* The fastest host $H_{1}$ is considered as the *low priority host* and is assigned a lower target throughput than the other hosts that are assigned the same throughput. This scenario intends to examine the simultaneous requests of the low throughput and the fairness among the hosts.

*(5) Low Priority Host B:* The same throughput setup is considered here except for the condition that the target throughput for $H_{1}$ is considered as the *minimum target throughput*.

### 5.3. Throughput Results

**Figure 5:**
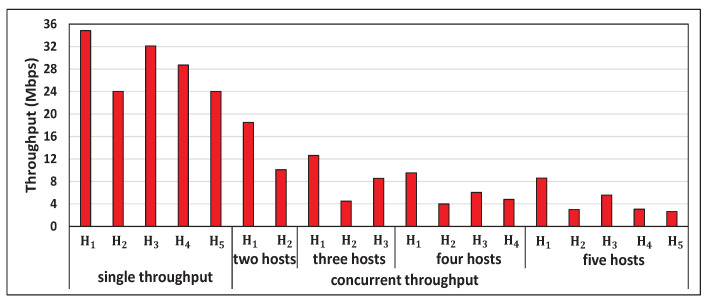
Measurement results of single and concurrent throughputs.

**Figure 6:**
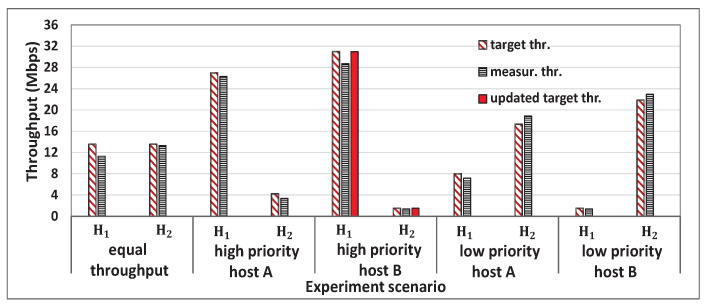
Results for two hosts case with a proposal.

**Figure 7:**
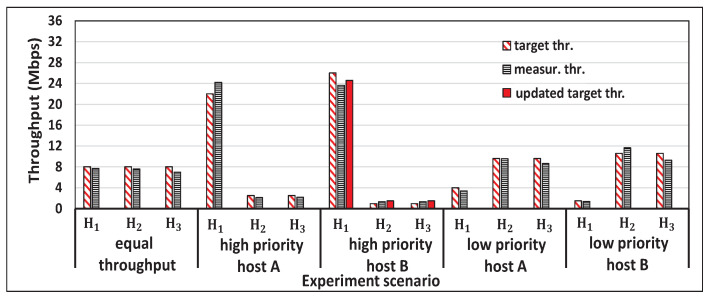
Results for three hosts case with a proposal.

**Figure 8:**
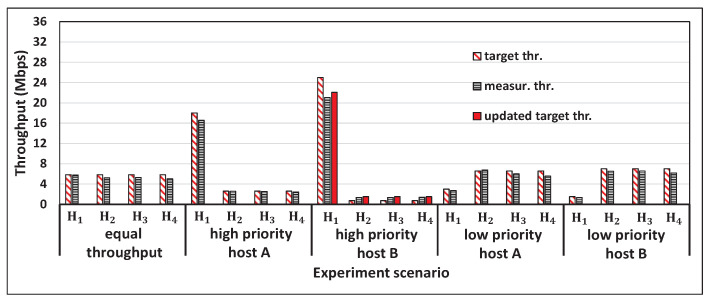
Results for four hosts case with a proposal.

**Figure 9:**
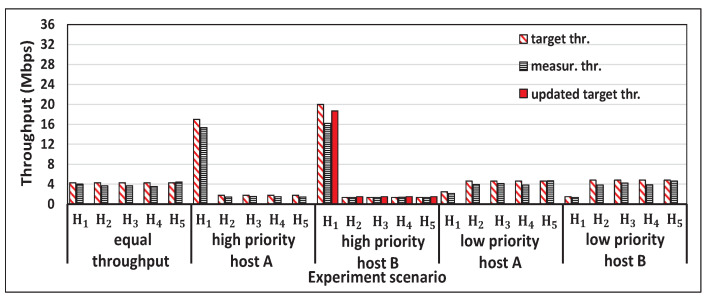
Results for five hosts case with a proposal.

Figure 5 shows the single throughput measurement results for the five hosts and the concurrent results for two, three, four, and five host cases with *iperf* traffic. Figure 6, Figure 7, Figure 8 and Figure 9 show individual host throughput results for concurrently communicating two, three, four, and five host cases, respectively. In each graph, *target thr.* represents the derived target throughput by the proposal and *measur. thr.* does the measured throughput. The *updated target thr.* indicates that the *Minimum Throughput Case* was applied there.

### 5.4. Discussions

From the experiment results, we observed the following results for these scenarios.

*(1) Equal Throughput:* The throughput unfairness occurs among the hosts when the proposal was not applied. This is because the closest host $H_{1}$ always achieves higher throughput than the other hosts. However, the measured throughput was similar among the hosts by assigning the equal target throughput by the proposal. Thus, the throughput fairness request was achieved by the proposal.

*(2) High Priority Host A:* The measured throughput of the *high priority host* $H_{1}$ always achieves the requested target throughput that was greater than its concurrent throughput, and the throughputs of the other hosts were similar to each other. However, the throughput of any host was greater than the *minimum target throughput*. Thus, both the high throughput request and the throughput fairness request were achieved.

*(3) High Priority Host B:* As in (2), both the high throughput request by of the *high priority host* $H_{1}$ and the throughput fairness request among other hosts were achieved. The original requested target throughput was updated, because it cannot ensure the the *minimum target throughput* for others.

*(4) Low Priority Host A:* The measured throughput of the *low priority host* always achieves the requested target throughput that was smaller than its concurrent throughput, and the throughputs of the other hosts were similar to each other. However, the throughput of any host was greater than the *minimum target throughput*. Thus, both the low throughput request for $H_{1}$ and the throughput fairness request among others were achieved.

*(5) Low Priority Host B:* As in (4), both the low throughput request and the throughput fairness request were achieved, while considering the *minimum target throughput* for $H_{1}$.

### 5.5. Fairness Index

To verify the throughput fairness for *equal throughput scenario*, Table 6 compares the *Jain’s fairness index* [33] of the measured throughput among the hosts. It shows that by applying the proposal, the fairness index is very close to 1.

## 6. Evaluations with Web Traffic

In this section, we evaluate the proposal through testbed experiments using web application traffic, instead of using practical experimentation. To generate high load traffic, the hosts are either downloading large files or accessing video streaming from websites.

### 6.1. Experimental Setup

**Figure 10:**
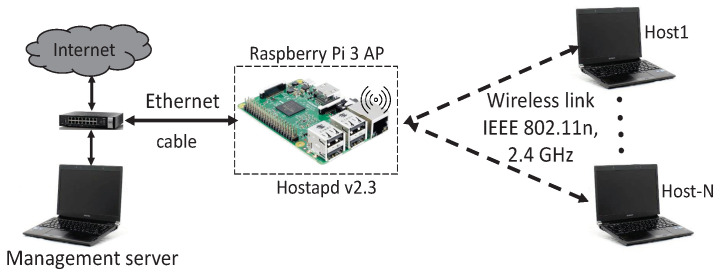
Testbed topology for Web traffic.

Figure 10 illustrates the network topology for the experiments using real web application traffic. As the web application servers in the Internet, *Ubuntu 20.04.3 OS* for file downloading [34] and *YouTube* for video streaming are adopted.

In the experiments, the number of hosts is increased from two to four, where the same devices and locations in Table 3 and Table 4 are used. Similarly, each experiment was conducted for 12 min.

The following three scenarios of *Equal Throughput*, *Priority Host*, and *Saturated Host* are examined, where the measured throughput is compared with and without applying the proposal.

*(1) Equal Throughput Scenario:* All the hosts are concurrently downloading the *Ubuntu 20.04.3 OS* files with 2.9 GB using the web browser from the *web server*. The *equal target throughput* is assigned to these hosts.

*(2) Priority Host Scenario:* All the hosts are concurrently downloading the *Ubuntu 20.04.3 OS* files. To investigate the effectiveness of the proposal, the slowest host $H_{2}$ is considered as the *priority host* and is assigned a far higher target throughput than the other hosts. This higher target throughput of the slowest host can be achieved by sacrificing the non priority hosts.

*(3) Saturated Host Scenario:* One host $H_{3}$ is streaming video using the web browser, and the other hosts are concurrently downloading the *Ubuntu 20.04.3 OS* files. Then, $H_{3}$ is considered as the *saturated host* that cannot utilize all the available bandwidth since its application requires the much smaller one. Then, the remaining bandwidth should be allocated to the other hosts equally.

### 6.2. Results for Equal Throughput Scenario

**Figure 11:**
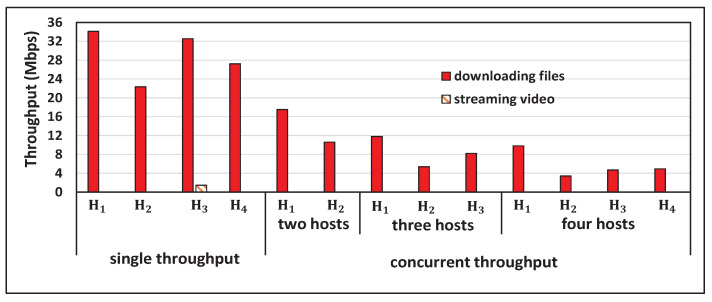
Measurement results of single and concurrent throughputs with web traffic.

**Figure 12:**
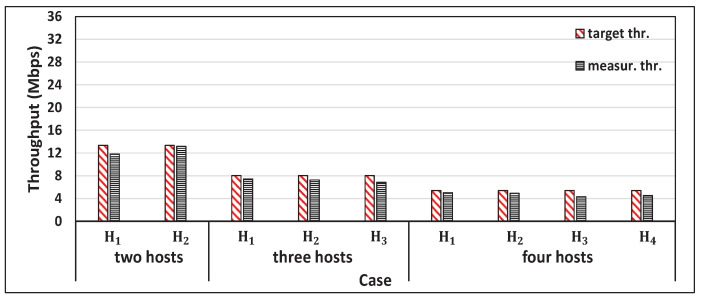
Results for *Equal Throughput Scenario* with proposal.

Figure 11 shows the single throughput measurement results for the four hosts and the concurrent results for two, three, and four host cases with web traffic. Figure 12 shows the target throughput and the measured throughput for two, three, and four hosts cases. When the proposal was not applied, the throughput unfairness appeared, where the near host from the AP, $H_{1}$, achieved a higher throughput than the others. On the other hand, when the proposal was applied, the similar measured throughput was achieved for all the hosts regardless of their locations. Table 7 compares the fairness index of the measured throughputs among the hosts with and without the proposal. The proposal increases the fairness index to be close to 1. Thus, the effectiveness of the proposal in solving the throughput unfairness problem is confirmed.

### 6.3. Results for Priority Host Scenario

**Figure 13:**
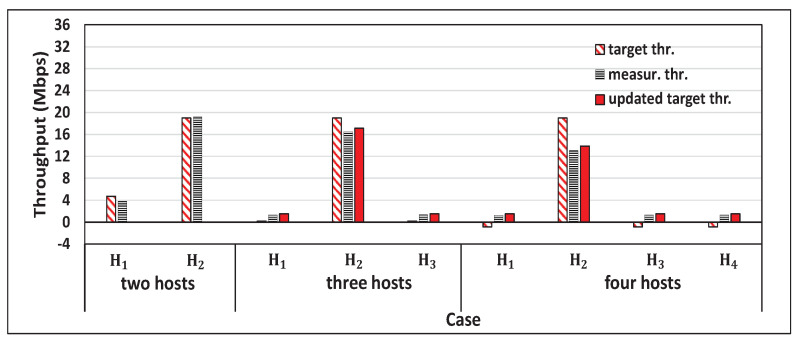
Results for *Priority Host* Scenario with proposal.

Figure 13 shows the results for *Priority Host* Scenario. Here, $H_{2}$ was selected as the priority host, because it was most distant from the AP. In three and four hosts cases, the target throughput was updated, because the original target throughput for $H_{2}$ cannot ensure the *minimum target throughput (1.5 Mbps)* of the others. Then, the proposal achieved the target throughput for any host.

### 6.4. Results for Saturated Host Scenario

**Figure 14:**
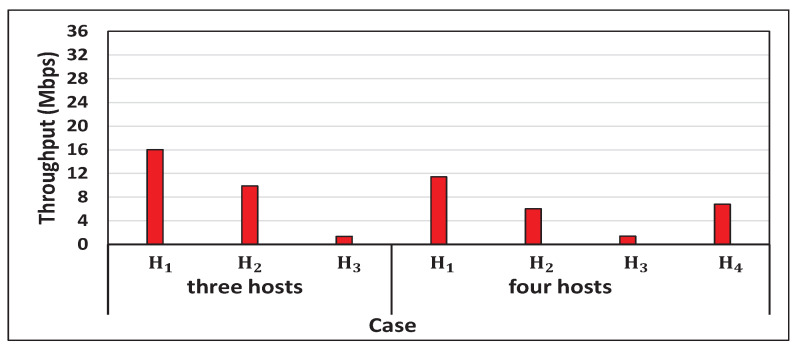
Measurement results of concurrent throughputs with *saturated host*.

**Figure 15:**
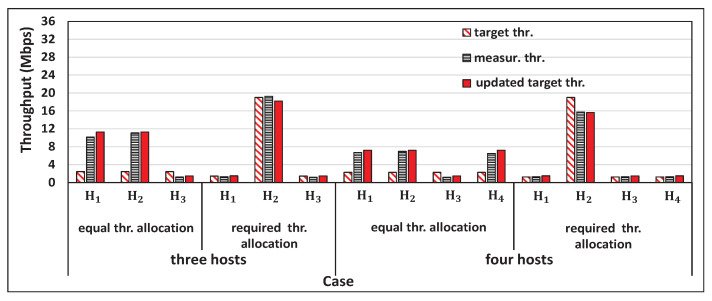
Results for *Saturated Host* with proposal.

Figure 14 shows the concurrent throughput measurement results for three and four host cases with the *saturated host* $H_{3}$. $H_{3}$ received the video streaming service, and was located in the same room as the AP. Figure 15 shows the results for *Saturated Host* Scenario. Two hosts’ case was not examined because only one host remained other than the saturated host. The measured single throughput for $H_{3}$, $S_{3}=1.47$ Mbps, is smaller than the obtained equal target throughput, 2.43 Mbps for three hosts case and 2.27 Mbps for four hosts’ case. Thus, $S_{3}$ was used for the target throughput of $H_{3}$, and the target throughput for the other hosts was updated. Then, the proposal achieved the target throughput for any host.

### 6.5. Throughput Comparison between the Proposal and without Proposal

**Figure 16:**
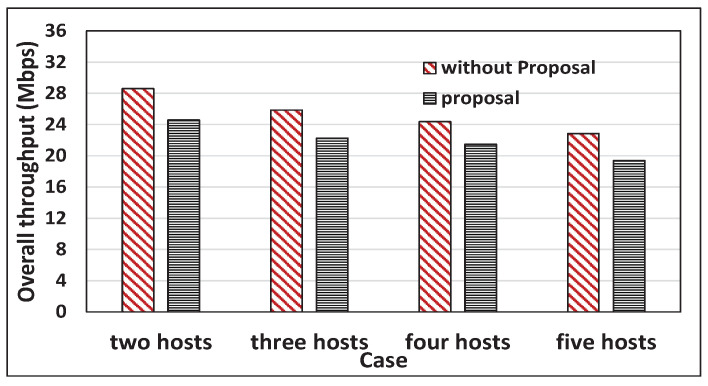
Total throughput comparisons for with and without proposal for *iperf* traffic.

**Figure 17:**
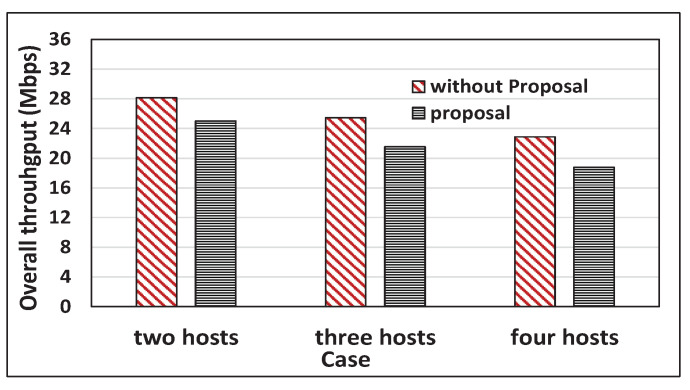
Total throughput comparisons for with and without a proposal for web traffic.

Figure 16 and Figure 17 compare the total throughput between the cases with the proposal and without the proposal. With the proposal, the total throughput is reduced by 14.36% and 14.77% on average for *iperf* and *web* traffic, which is tolerable. The packet transmissions with high bit rates to near hosts become reduced. The total throughput reduction cannot be avoided in achieving throughput fairness by giving more packet transmissions with low bit rates to distant hosts.

## 7. Conclusions

This paper proposed the *throughput request satisfaction method* for concurrently communicating multiple hosts with a single access point (AP) in a wireless local area network (WLAN). To meet the fair or necessary throughput request, the method measures the single and concurrent throughput for each host, calculates the *channel occupying time*, derives the target throughput to satisfy the request, and controls the traffic to achieve the target throughput of every host by applying *traffic shaping* at the AP.

For evaluations, the method was implemented on the WLAN testbed system with one *Raspberry Pi* AP and up to five hosts. The extensive experiment results in five scenarios confirmed that the proposal achieved fair throughput by allocating the equal throughput, and the required throughput of the host. Further, the proposal was evaluated using web traffic for real applications and was confirmed to work well.

In future studies, we will extend the proposal to consider multiple APs and host mobility in the network where hosts may frequently join or leave the network. Besides, we will also study the throughput enhancement at the increasing throughput fairness. Then, we will evaluate our proposals in various network fields and topologies to confirm their effectiveness.

## Figures and Tables

**Table 1:** Overview of related works.

Reference	Goal	Approach and Implementation	Advantage	Disadvantage and Non Solved Problem	Evaluation
proposal	throughput fairness per host	throughput control by traffic shaping, implemented by the Linux command	does not require any modification of hardware or protocol	reduce the overall throughput	testbed experiment
[10]	throughput fairness per flow	uplink/downlink flow control by modified backoff algorithm in MAC layer.	increase the overall throughput	modification of MAC layer protocol and cannot allocate equal or required throughput	simulation
[11]	fairness between uplink/downlink TCP flows	dual queue for optimum queue selection in MAC layer	increase the overall throughput	modification of MAC layer protocol and cannot allocate equal or required throughput	simulation
[12]	throughput fairness per flow	DCF enhancement by HARQ in MAC layer	increase the overall throughput	modification of MAC layer protocol and cannot allocate equal or required throughput	simulation
[13]	airtime fairness	queue management based on data rates, which is implemented in MAC layer	increase the overall throughput	modification of MAC layer protocol and cannot allocate equal or required throughput	simulation
[14]	airtime fairness	packet scheduling and channel access control, which is implemented on AP	increase the overall throughput	modification of AP and cannot allocate equal or required throughput	simulation
[15]	airtime fairness	airtime control by traffic shaping, implemented by the Linux command	increase the overall throughput	cannot allocate equal or required throughput	testbed experiment
[16]	max-min fairness	network-level queueing prioritization, implemented by the Linux command	increase the user level QoS by allocating throughput	cannot allocate equal required throuhgput	testbed experiment
[17]	throughput allocation	throughput control by traffic shaping, implemented by ns-2 simulator	provide throughput fairness	cannot allocate necessary throughput, and reduce the overall throughput	simulation
[18]	airtime fairness	network-level queue management, implemented in Linux kernel	increase the overall throughput	cannot allocate equal or required throughput	testbed experiement
[19]	airtime fairness	changing contention window by modifying MAC protocol	increase the overall throughput	modification of MAC layer protocol and cannot allocate equal or required throughput	simulation
[20]	airtime fairness	queueing prioritization, implemented by the Linux command	increase the overall throughput	cannot allocate equal or required throughput and hardware specific approach	testbed experiment
[21]	airtime fairness	packet scheduling algorithm in MAC layer	increase the overall throughput	cannot allocate equal or required throughput and modification of scheduler	simulation
[22]	fairness among virtual WLANs	contention window adjustment by PI controller	increase the overall throughput	cannot allocate equal or required throughput and modification of hardware	simulation
[23]	throughput fairness	DCF enhancement by HARQ, Implemented in MAC layer	increase the overall throughput	cannot allocate equal or required throughput and modification of hardware	simulation
[24]	airtime fairness	SDN approach, and bandwidth control by traffic shaping	increase the overall throughput	cannot allocate equal or required throughput	testbed experiment

**Table 2:** Definition of notations.

Notations	Definition
$H_{i}$	i^th^ host for i=1,2,...,n, where *n* is the number of host
$S_{i}$	measured *single throughput* of $H_{i}$
$C_{i}$	measured *concurrent throughput* of $H_{i}$
$t_{i}$	*target throughput* of $H_{i}$
$t_{min}$	*minimum target throughput* for any host
$d_{i}$	rate and ceil parameter value
$R_{i}\left(m\right)$	measured throughput at time step *m*

**Table 3:** Hardware and software specifications.

Server and Hosts	
type	1. Toshiba Dynabook R731/B,
	2. Toshiba Dynabook R734/K
operating system	Linux (Ubuntu 14)
processor	1. Intel Core i5-2520M, 2.5 GHz
	2. Intel Core i5-4300M @2.6 GHz
RAM	4GB DDR3-1333 MHz
software	*iperf 2.0.5*
access point	
type	Raspberry Pi 3
operating system	Linux (Raspbian)
processor	BCM2837 1.2 GHz, Broadcom
RAM	LPDDR2 900 MHz 1GB
NIC	BCM43438, Broadcom
software	*hostapd, iftop*

**Table 4:** Device locations.

Case	Device Location		
	D307	in front of D307	Refresh Corner
2 hosts	AP, $H_{1}$	–	$H_{2}$
3 hosts	AP, $H_{1}$, $H_{3}$	–	$H_{2}$
4 hosts	AP, $H_{1}$, $H_{3}$	$H_{4}$	$H_{2}$
5 hosts	AP, $H_{1}$, $H_{3}$	$H_{4}$	$H_{2}$, $H_{5}$

**Table 5:** Target throughput conditions in five scenarios.

Scenario	Condition
(1) equal throughput	$t_{1}=t_{2}=t_{3}=t_{4}=t_{5}$
(2) high priority host A	$t_{1}>t_{i}$ and $t_{i}>t_{min}$
(3) high priority host B	$t_{1}>t_{i}$ and $t_{i}<t_{min}$
(4) low priority host A	$t_{1}<t_{i}$ and $t_{i}>t_{min}$
(5) low priority host B	$t_{1}<t_{i}$ and $t_{1}=t_{min}$

**Table 6:** Fairness index comparison for *equal throughput scenario*.

Case	Fairness Index	
	without Proposal	Proposal
2 hosts	0.920	0.993
3 hosts	0.869	0.998
4 hosts	0.842	0.995
5 hosts	0.802	0.991

**Table 7:** Fairness index comparison for *equal throughput*.

Case	Fairness Index	
	without Proposal	Proposal
2 hosts	0.942	0.996
3 hosts	0.912	0.998
4 hosts	0.847	0.996

## Data Availability

Not applicable.
